# Cardiac resynchronization therapy guided by the new KODEX‐EPD imaging system

**DOI:** 10.1002/joa3.12627

**Published:** 2021-08-30

**Authors:** Antonio Scarà, Luigi Sciarra, Edoardo Bressi, Ermenegildo De Ruvo, Domenico Grieco, Alessio Borrelli, Paolo Zecchi, Leonardo Calò

**Affiliations:** ^1^ Policlinico Casilino Hospital Rome Italy; ^2^ NS della Mercede Hospital Rome Italy

**Keywords:** biventricular, CRT‐D, electroanatomic mapping system, Kodex‐EPD

## Abstract

Nowadays, fluoroscopy is the standard tool used to help physicians during pacing lead implantation. However, its use entails significant radiation exposure for physicians and especially for patients. For the first time, the present case report describes the use of the electro‐anatomical mapping (EAM) navigation system KODEX‐EPD for cardiac resynchronization therapy (CRT) implantation. These findings suggest that CRT implantation guided by the KODEX‐EPD system is feasible and safe with the minimization of X‐ray and dye exposure.

## Graphical abstract

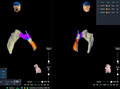

## 1 BACKGROUND

Nowadays, fluoroscopy is the standard tool used to help physicians during pacing lead implantation. However, its use entails significant radiation exposure for physicians and especially for patients. In this scenario, device implantation guided by EAM systems is an emerging approach, providing combined information on anatomy and activation patterns and reducing fluoroscopy and angiography times.^1^ Moreover, EAM‐guided procedure could be helpful, especially in patients with heart failure undergoing CRT where iodine contrast medium infusion is required for coronary sinus (CS) branch visualization. We, therefore, present the first case of CS lead implantation guided by EAM system KODEX‐EPD.

## 2 OBJECTIVE

The purpose of this case report is to describe the usefulness of EAM‐guided lead implantation with a new dielectric imaging system in a patient allergic to dye injection undergone CRT‐D.

## 3 METHODS

### 3.1 Patient and Kodex system characteristics

A 72‐year‐old woman with nonischemic cardiomyopathy (NICM), severe left ventricular dysfunction (LVEF 30%), left bundle branch block (LBBB), New York Heart Association (NYHA) class III, was selected for CRT implantation. In her clinical history, a severe allergic reaction to iodine contrast medium was reported. Therefore, we decided to use the KODEX‐EPD imaging system, version 1.4.7 (EPD Solutions, Philips, Best, The Netherlands) to perform the procedure avoiding contrast medium exposure.

The KODEX‐EPD system (EPD Solutions) offers guidance via high‐definition (HD)‐computed tomography such as cardiac imaging and visualization of the cardiac chambers with navigation spatial resolution of 0.27 mm. Compared with the NavX Ensite precision and CARTO system, the Kodex system allows to build the anatomy with a new technology based on dielectric‐sensing acquiring anatomy 1 cm ahead of the electrodes; the system recognizes the bending of the electric field due to blood vessel steep field gradients. In addition, it is an open system and does not need dedicated or branded catheters; imaging and mapping can be done with any catheter with electrodes including pacing leads.

### 3.2 Procedure description

An external double left subclavian puncture was performed using anatomic references. A single‐coil active fixation lead and a bipolar active fixation lead were positioned in the right ventricle and right atrial appendage, respectively.

**FIGURE 1:**
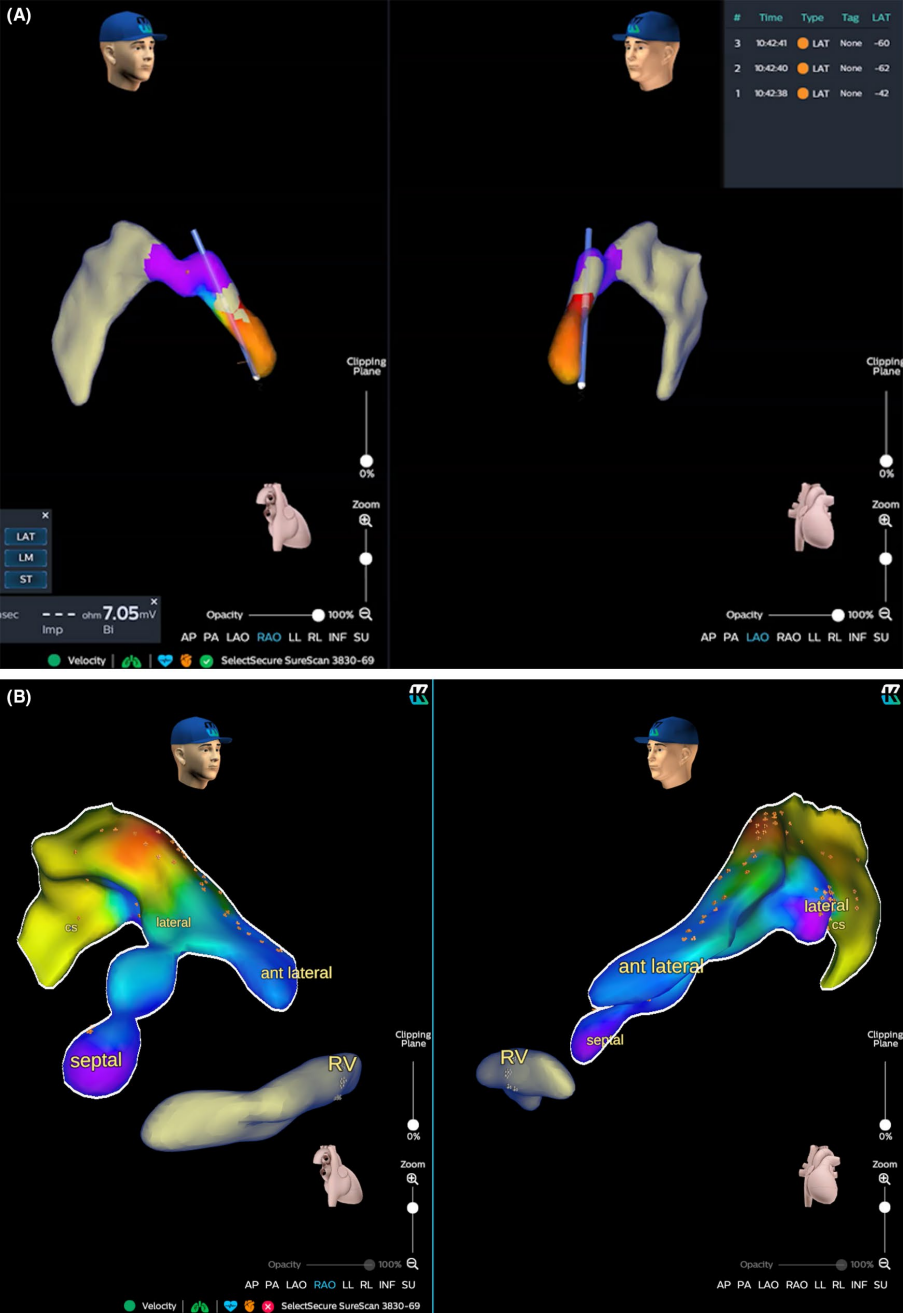
(A) Pacing lead displayed on KODEX‐EPD system in the initial phases of imaging and mapping of CS and branches. (B) Bipolar map of the coronary sinus. Three branches are represented (lateral, anterolateral, and septal). Low voltages were recorded in lateral and anterolateral branches (displayed in yellow and light*‐*blue). Normal voltages were found in the septal branch (displayed in violet)

**FIGURE 2:**
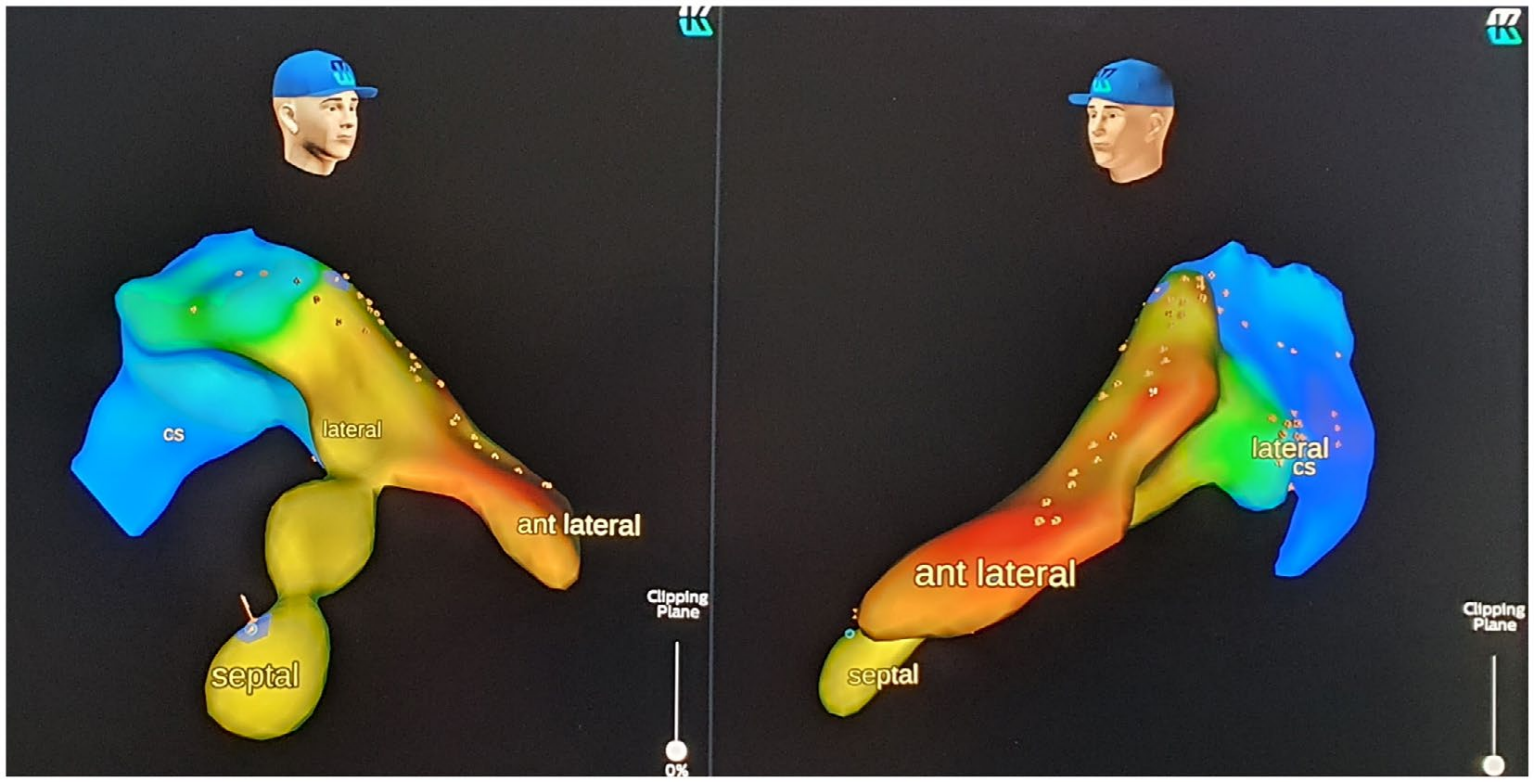
Activation map (LAT) of the coronary sinus. Three branches are represented (lateral, anterolateral, and septal). The latest activation was found in the lateral branch (displayed in light*‐*green) but with an elevated pacing threshold; for this reason, as the second choice, the septal branch (displayed in yellow) was selected for the final catheter position (flag)

After that, a quadripolar electrophysiology catheter was connected to the Kodex‐EPD system employing pacing threshold cables with crocodile clips and was introduced via the delivery system through the left subclavian vein. The entire system was used to track the right ventricular septal anatomy and cannulate the coronary sinus without an X‐ray. Thus, the pacing CRT lead (Medtronic Attain Performa MRI Sure Scan; 5 French), using the over‐the‐wire technique connected to the imaging system, allowed us to explore the branches, acquire the local intracardiac signal, and reach the target site. In particular, it allowed us to detect the coronary sinus branches without using contrast liquid infusion and fluoroscopy. During the coronary sinus mapping, the local activation time map was acquired to investigate the local electrical delay from the onset of QRS, and bipolar voltages were acquired for any branch explored to predict stimulation threshold (Figure 1). Three veins were initially imaged: a septal vein, an anterior vein, and an anterolateral vein. Anterior and anterolateral veins portions were navigated and depicted as distal as possible using the materials described, but not stable catheter positions have been reached, neither adequate pacing thresholds. Therefore, the final catheter position was chosen following the best combination of local activation delay^2^ (Figure 2) with an acceptable pacing threshold (0.75 mV at 0.5 ms). Finally, leads were connected to the device (Medtronic Amplia CRT‐D).

## 4 RESULTS

The total procedure time was 80 minutes, while fluoroscopy time was 2 minutes.

**FIGURE 3:**
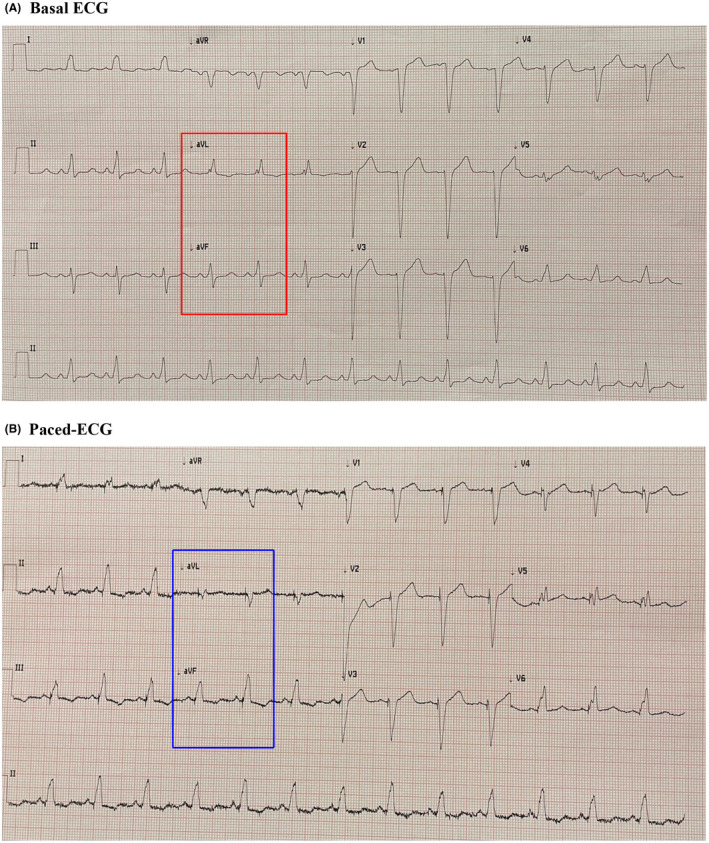
(A) Basal ECG: spontaneous QRS duration 155 ms, left axis deviation (see red rectangle). (B) Paced QRS: QRS duration 120 ms, normal‐oriented axis (see blue rectangle)

Fluoroscopy use was minimized and limited to instantly check for a left subclavian puncture, follow the single‐coil lead and atrial lead positioning, help CS cannulation, and confirm lead for the left ventricle (LV) position in the different CS branches during the procedure. Lead implantation was performed targeting different anatomical regions and using KODEX‐EPD for mapping and lead navigation. Indeed, KODEX‐EPD has been used for mapping better stimulation sites, especially for coronary sinus branches with the pacing catheter and without any add‐ons. Nonetheless, the KODEX‐EPD‐guided procedure did not require any change in the procedure flow: the pacing lead has been used to image and guide the whole procedure, while no additional electrophysiology catheter needed to be employed. The finally obtained biventricular paced‐QRS was 120 ms duration (vs. spontaneous QRS duration 155 ms) (Figure 3A,B). Moreover, thanks to the electro‐anatomical guidance to reach the target vein, in this case, we appreciated a correction of basal left axis deviation that changed in a normal‐oriented axis after pacing (Figure 3A,B).

## 5 CONCLUSION

The present case report firstly describes the use of the EAM navigation system KODEX‐EPD for CRT implantation. These findings suggest that CRT implantation guided by the KODEX‐EPD system is feasible and safe with the minimization of X‐ray and dye exposure.

## 6 LIMITATIONS

The anatomical resolution of images acquired by KODEX‐EPD is still limited because the system builds the anatomy with imaging technology and manual editing of the anatomy is not possible (no shaver or eraser are created in the system). However, additional features of the system are under development to allow more detailed anatomical reconstructions.

## CONFLICT OF INTEREST

All authors have no conflict of interest to declare.
